# Evaluation of Exposure Assessment Methods and Procedures for Induction Hobs

**DOI:** 10.1002/bem.70024

**Published:** 2025-09-29

**Authors:** Jingtian Xi, Sven Kühn, Cosimo Fortunato, Erdem Ofli, Niels Kuster

**Affiliations:** ^1^ IT'IS Foundation Zurich Switzerland; ^2^ ZMT Zurich MedTech AG Zurich Switzerland; ^3^ Department of Information Technology and Electrical Engineering Swiss Federal Institute of Technology (ETH Zurich) Zurich Switzerland

**Keywords:** compliance evaluation, contact currents, exposure assessment, incident field, induced field, induction hobs

## Abstract

Induction hobs generate strong alternating magnetic fields to heat pots by inducing eddy currents. These fields are the strongest close to the bottom of the cookware, but stray fields at large distances can still be substantial. In general, these are higher than the reference levels defined by international electromagnetic exposure safety guidelines (ICNIRP 1998; ICNIRP 2010; IEEE 2019). That the reference levels are exceeded does not imply that the basic restrictions are also violated. In this study, we assess the exposures caused by the latest generation of induction hobs by applying the advanced instrumentation and different methods that include the procedures developed by the International Electrotechnical Commission (IEC) for household appliances (IEC 62233) (IEC International Electrotechnical Commission 2005), the 4‐tier approach developed for inductive wireless power transfer systems (IEC 63184) (IEC International Electrotechnical Commission 2021), and their derivatives. First, methods for determining the maximum exposure configuration were assessed. Then, the 3D distribution of the incident magnetic field was sampled with a scanning system and analyzed, and the contact currents assessed. Lastly, numerical dosimetric evaluations were performed in anatomical models to determine the maximum fields induced by the measured incident fields directly or by a representative coil model converted from the measured fields. The study's findings reveal significant variations in exposure across different induction hobs, with differences of up to a factor of > 20 (> 26 dB) as a function of power, coil size, and proximity to the coil. This suggests that low‐exposure hobs can be designed without compromising cooking performance. Furthermore, the study strengthens the conclusions of previous studies that IEC 62233 (IEC International Electrotechnical Commission 2005) may underestimate the exposure for persons standing next to the hob by up to a factor of > 30—based on testing according to the exposure limits from (ICNIRP 1998; IEEE 2019)—and thus does not ensure safety. A dosimetric analysis, the most accurate method, would be relatively costly. Alternative approaches derived from (IEC International Electrotechnical Commission, 2021) that are affordable and not overly conservative are discussed. Bioelectromagnetics. 00:00–00, 2025. © 2025 Bioelectromagnetics Society.

## Summary

- The low‐exposure induction hobs can be achieved by employing small heating coils without compromising cooking performance.
- The product standards IEC 62233 (IEC International Electrotechnical Commission 2005) and EN 62233 (CENELEC, European Committee for Electrotechnical Standardization 2008) may significantly underestimate the exposure, and therefore require revision to ensure the safety of induction hobs.
- Conservative and affordable approaches from IEC 63184 (IEC International Electrotechnical Commission 2021) and IEC 60990 (IEC International Electrotechnical Commission 2016) are applied to demonstrate the compliance of induction hobs with the limits of incident/induced fields and contact current, respectively.

## 1 Introduction

Induction cooking is based on the principle of magnetic induction: an oscillating magnetic (*H*−) field induces eddy currents in cookware, leading to Joule heating (Lucía et al. 2013). Further heating is generated by magnetic hysteresis in ferromagnetic materials. The typical operation frequency range of the induction hobs is 20–100 kHz. Compared to other cooking appliances, such as gas ranges or conventional electric cook‐tops, induction hobs have a number of advantages; for example, time and energy savings, controlled heat supply, reduced risk of severe burns, and easier cleaning (Sweeney et al. 2014).

However, since the *H*‐field cannot be fully confined to the cookware, regulators have raised concerns about the compliance of the induction hobs with human exposure limits. These electromagnetic (EM) exposure limits have been defined by different organizations, for example, the International Commission on Nonionizing Radiation Protection (ICNIRP) (ICNIRP 1998, 2010) and the Institute of Electrical and Electronics Engineers (IEEE) (IEEE 2019). Currently, most countries, including the European Union (EU) nations (European Council 1999) and Switzerland, have adopted the earlier ICNIRP 1998 guidelines. The latest ICNIRP 2010 guidelines (ICNIRP 2010) offer relaxations of the limits over some frequency ranges that have not yet been passed into law. The guidelines/standards define reference level (RL) limits for incident fields and basic restriction (BR) limits for induced fields (e.g., induced electric (*E*−) fields, induced current densities *J*). The assessment of exposure compliance with RL is easier but much more conservative than the assessment based on BR limits, as the RL has been determined for uniform field exposures. However, the fields of induction hobs are not uniform, highest close to the heating coil and decaying rapidly with distance. The applicable limits for induction hobs are summarized in Table 1. In its 2010 guidelines, the ICNIRP changed the BR metric from induced current density of (ICNIRP 1998) to induced *E*‐field (ICNIRP 2010) to account for nerve stimulation not only in the central nervous system but also in the peripheral nervous system. It should be noted that stray quasi‐static incident *E*‐fields can be relatively large and exceed the relevant RL limits. However, the conducted current assessment is a sufficiently robust method to demonstrate compliance with the contact current limits.

**Table 1:** Applicable electromagnetic safety limits in the frequency range 20–100 kHz.

Guidelines/Standard	RL or BR	Quantity	Value	Remarks
ICNIRP 1998 ICNIRP (1998)	RL	*H*	5 A/m	
ICNIRP 1998 ICNIRP (1998)	BR	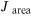	$2\cdot f_{\text{MHz}}\;{\;\text{A/m}\;}^{2}$	Averaged over $1\;{\;\text{cm}\;}^{2}$
ICNIRP 2010 ICNIRP (2010)	RL	*H*	21 A/m	
ICNIRP 2010 ICNIRP (2010)	BR	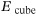	$135\cdot f_{\text{MHz}}$ V/m	Averaged over $8\;{\;\text{mm}\;}^{3}$
IEEE Std. C95.1‐2019 IEEE (2019)	RL	*H*	163 A/m	Valid for head/torso
IEEE Std. C95.1‐2019 IEEE (2019)	RL	*H*	900 A/m	Valid for limbs
IEEE Std. C95.1‐2019 IEEE (2019)	BR	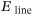	$209\cdot f_{\text{MHz}}$ V/m	Averaged over 5 mm, valid for other tissues
				(i.e., except brain, heart, or limbs)
IEEE Std. C95.1‐2019 IEEE (2019)	BR	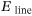	$627\cdot f_{\text{MHz}}$ V/m	Averaged over 5 mm, valid for limbs

*Note:* The limits for the incident magnetic field (*H*), the induced current density (*J*), and the induced electric field (*E*) listed here are all instantaneous and root‐mean‐square (rms) values. The limits of IEEE and ICNIRP are based on different simplified human phantoms and an acute hazard model for instantaneous nerve excitation with different safety factors. It should be further noted that the biological basis for the spatial averaging schemes is weak.

The International Electrotechnical Commission (IEC) Technical Committee (TC) 106 develops general and product‐specific standards to demonstrate compliance with the RL and/or BR limits. The standards relevant for the frequency range of induction hobs are IEC 62233:2005 (IEC International Electrotechnical Commission 2005) and the European Standard EN 62233:2008 (CENELEC, European Committee for Electrotechnical Standardization 2008), which is identical to IEC 62233:2005 as far as measurement methods are concerned. The most recent advances in exposure assessment instrumentation facilitate more comprehensive assessment methods and procedures for inductive wireless power transfer (WPT) systems as described in IEC 63184 (IEC International Electrotechnical Commission 2021), the frequency range (3 kHz–10 MHz) of which overlaps with the operation frequencies of induction hobs. In the 4‐tier approach of (IEC International Electrotechnical Commission 2021), Tier 2 is a method for assessment of the incident field at all locations where a person may be exposed with comparison to the RL limits. As these fields decay steeply as a function of distance, Tier 2 is usually very conservative. With Tier 3, the gradient of the incident field is used to estimate the induced fields based on the generic gradient source model (GGSM) (Liorni et al. 2020), resulting in a much lower overestimation of the exposure. Tier 4, the most accurate way to demonstrate compliance with the BR limits, requires a volume scan of the field or a validated source model and a number of simulations covering the possible exposure scenarios.

The overall objective of this study is to evaluate comprehensive but feasible methods and procedures to determine compliance of induction hobs with EM safety limits. Our approach is divided into four steps, each of which has its own method and result sections:

- *Methods for determination of the maximum exposure configuration:* The exposure depends not only on the power level but also on the characteristics of the pot (e.g., size, material, etc.) and its position with respect to the heating coil and the person. We evaluated methods for determination of the maximum exposure configuration with reasonable effort.
- *Methods for incident field assessment:* The incident field is measured within a sufficiently fine grid that encompasses the volume where a person may be exposed and can be used for demonstrating compliance with the RL. The incident field can also be used as input for dosimetric assessment in anatomical models representing persons standing next to the hob.
- *Methods for determining the maximum contact currents:* The maximum contact currents are measured according to variants of the standardized procedure (IEC International Electrotechnical Commission 2016).
- *Methods for determining the maximum induced fields in exposed persons:* The maximum induced fields are evaluated in a representative set of anatomical models for comparison with the BR.

In the discussion, we explore approaches to the determination of compliance in a robust and conservative manner that requires reasonable levels of effort. These findings may provide valuable input for the revision of the product standard.

## 2 Devices Under Test

Based on a Swiss market review of the most recent induction hobs, we distinguish four categories:

- induction hobs with fixed heating zones.
- induction hobs with a bridging feature that enables multiple heating zones to function as a unified single heating zone.
- induction hobs with both fixed heating zones and bridged zones, that is, a hybrid of types 1 and 2.
- induction hobs with full‐flex function that allows users to place cookware at any location within the full‐flex heating area.

Four hobs representing the latest technologies were selected for the study, see Table 2. The heating capacities of the induction hobs are listed in Table 2 as the maximum powers for a single pot, based on the values provided in the respective data sheets. For device D, however, only the maximum power consumption is listed as the maximum power for a single pot is not available. Overall, the heating capacities of the four hobs are comparable. The two flex‐zone hobs, device C and device D, consist of eight medium heating coils and 32 small heating coils, respectively.

**Table 2:** Maximum power, suggested cookware diameter, outer diameter of the heating coils, and features of the four representative hobs.

Device	Catergory	Max. power	Pot dia. suggested	Heating coil dia.	Features
		[kW]	[cm]	[cm]	
A	1	3.7	14.5–21	14.5, 18, 21	Boost power
B	2	3.2	12.5–21	22	Boost power, bridge function
C	4	3.65	14.5–35	17	Boost power, full‐flex function
D	4	7.4	≥10	7.4	Boost power, full‐flex function

The cookware set selected for the study consists of 19 pots of varying shapes (round, rectangular, and oval bottoms), materials (stainless steel, cast iron, cast aluminum, and titanium), and dimensions (diameters of 10–28 cm for round pots).

## 3 Evaluation of Maximum Exposure Configurations

### 3.1 Methods

**Figure 1:**
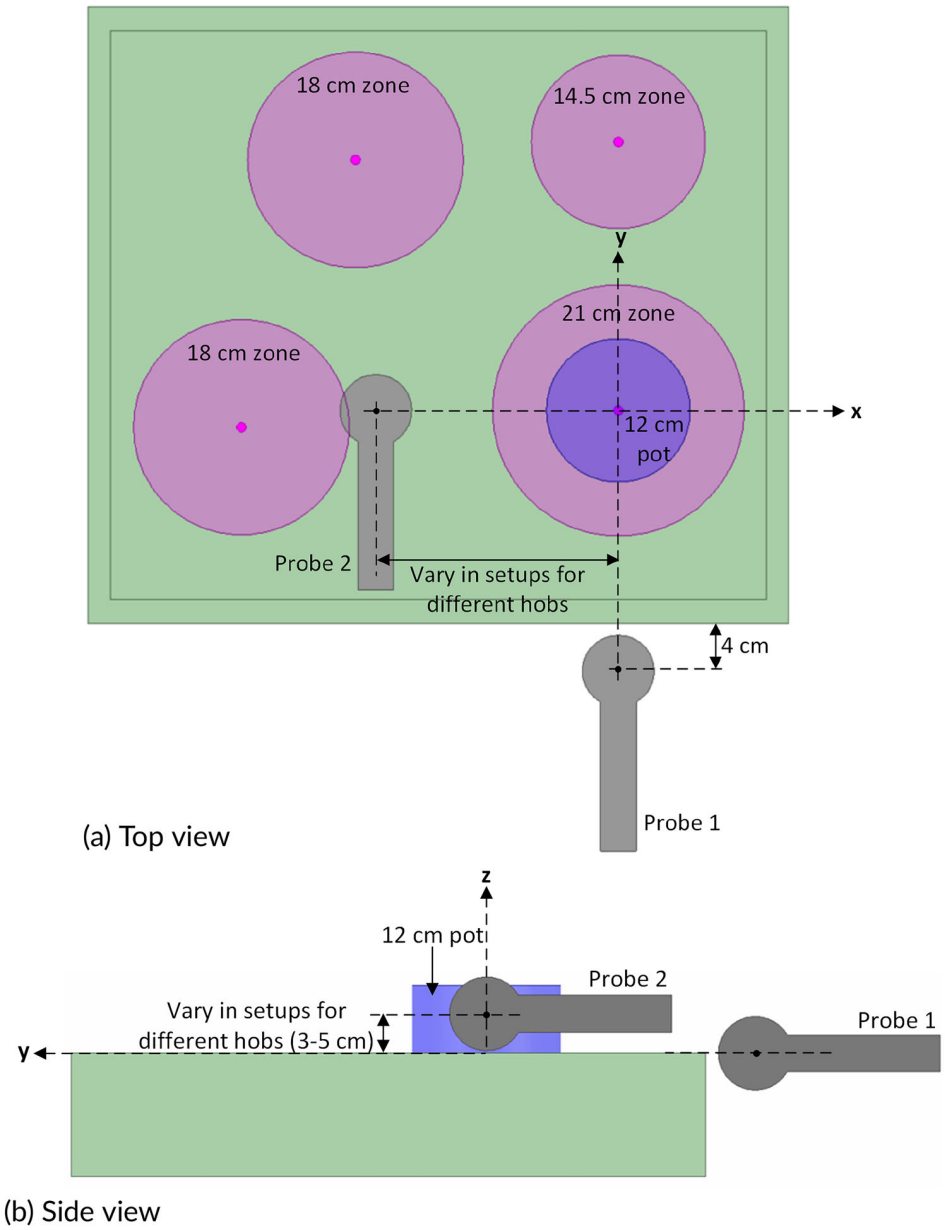
Placement of the two MAGPy probes in the evaluations of maximum exposure configurations, illustrated with device A as seen (a) from above and (b) from the side. (a) Top view; (b) Side view.

Two MAGPy (Schmid & Partner Engineering AG, Switzerland) probes were simultaneously used to identify the maximum exposure configurations. As shown in Figure 1, probe 1 was placed in front of the hob with a spacing of 4 cm between the center of the probe and the edge of the hob. The probe head was horizontally aligned (i.e., along the x axis) with the heating zone for fixed‐zone hobs or with the pot for flex‐zone hobs; the probe was vertically aligned (i.e., along the z axis) with the top surface of the hob. Probe 2 was placed above the hob and to the left of the heating zone (for fixed‐zone hobs) or the pot (for flex‐zone hobs). Probe 1 provides information about the field in front of the hob, and probe 2 provides information about the field in the vicinity of the heating coil. The influence of the following factors upon the *H*‐field amplitudes measured by the two probes was studied individually: (a) pot characteristics, (b) heating zone (applicable to fixed‐zone hobs), (c) pot offset (applicable to fixed‐zone hobs), (d) pot position (applicable to flex‐zone hobs), (e) power level, and (f) number of pots being heated simultaneously. For example, to investigate the influence of pot characteristics, only the pot was changed, while all the other factors and the probe positions were fixed. The *H*‐field amplitudes measured by the two probes for different pots were compared, and the pot that resulted in the maximum *H*‐field amplitudes (i.e., “the highest *H*‐field”) was recorded.

### 3.2 Results

The important observations from the survey are summarized here.

- Pot characteristics: Generally pots having the smallest bottom diameter and made of cast iron produce the highest *H*‐fields. Note that the 10 cm pot is too small to be detected in many cases, and as such is not taken into consideration as a potential worst‐case pot.
- Heating zone: The largest front heating zone produces the highest *H*‐fields. A larger heating zone is usually associated with a higher power rating.
- Pot centering relative to the heating zones of fixed‐zone hobs: The *H*‐fields measured with small pots are less sensitive to pot centering than are large pots. As an example, for the pot with diameter 12 cm on device A, an offset of up to 4 cm (beyond which the pot is not detected) leads to a maximum increase of 0.9 dB in the total *H*‐field.
- Pot position on the heating area of flex‐zone hobs: The worst‐case pot position corresponds to the activation of multiple heating coils in the flex‐zone hobs and the constructive interference of the *H*‐fields from the coils. For device C, the largest *H*‐field is observed when the pot is placed between two heating coils along the x‐axis (from left to right) and at the very front along the y‐axis (from front to back). For device D, the largest *H*‐field is found when the pot is centered within four heating coils.
- Power level: The *H*‐field always increases with the power level (note that the boost power level is not always available).
- Multiple‐pot operation: The heating of multiple pots shows small increases (<1.6 dB) in the *H*‐field on devices B–D. However, on device A, the use of two pots leads to an increase of about 4 dB due to the constructive interference of fields from multiple heating coils; adding more pots causes little further increase.

Accordingly, the maximum exposure configurations identified are:

- Device A with single‐pot operation: Pot‐S‐12 (12 cm diameter cast iron pot) on the 21 cm diameter heating zone; power level boost; no pot offset.
- Device A with multiple‐pot operation: the Pot‐S‐12 on the 21 cm heating zone and the Pot‐W‐14 (14 cm diameter stainless steel pot) on the 14.5 cm diameter heating zone; power level 9, which is the highest power level attainable when two heating zones on the same side are operated at the same time; no pot offset.
- Device B: Pot‐S‐12 on the left front heating zone; power level boost; no pot offset.
- Device C: Pot‐S‐12 touching the front edge of the heating zone and positioned between two activated coils; power level boost.
- Device D: Pot‐S‐12 placed in the center of four front heating coils; power level boost.

## 4 Evaluation of the Incident Fields and RL Quantities

### 4.1 Methods

**Figure 2:**
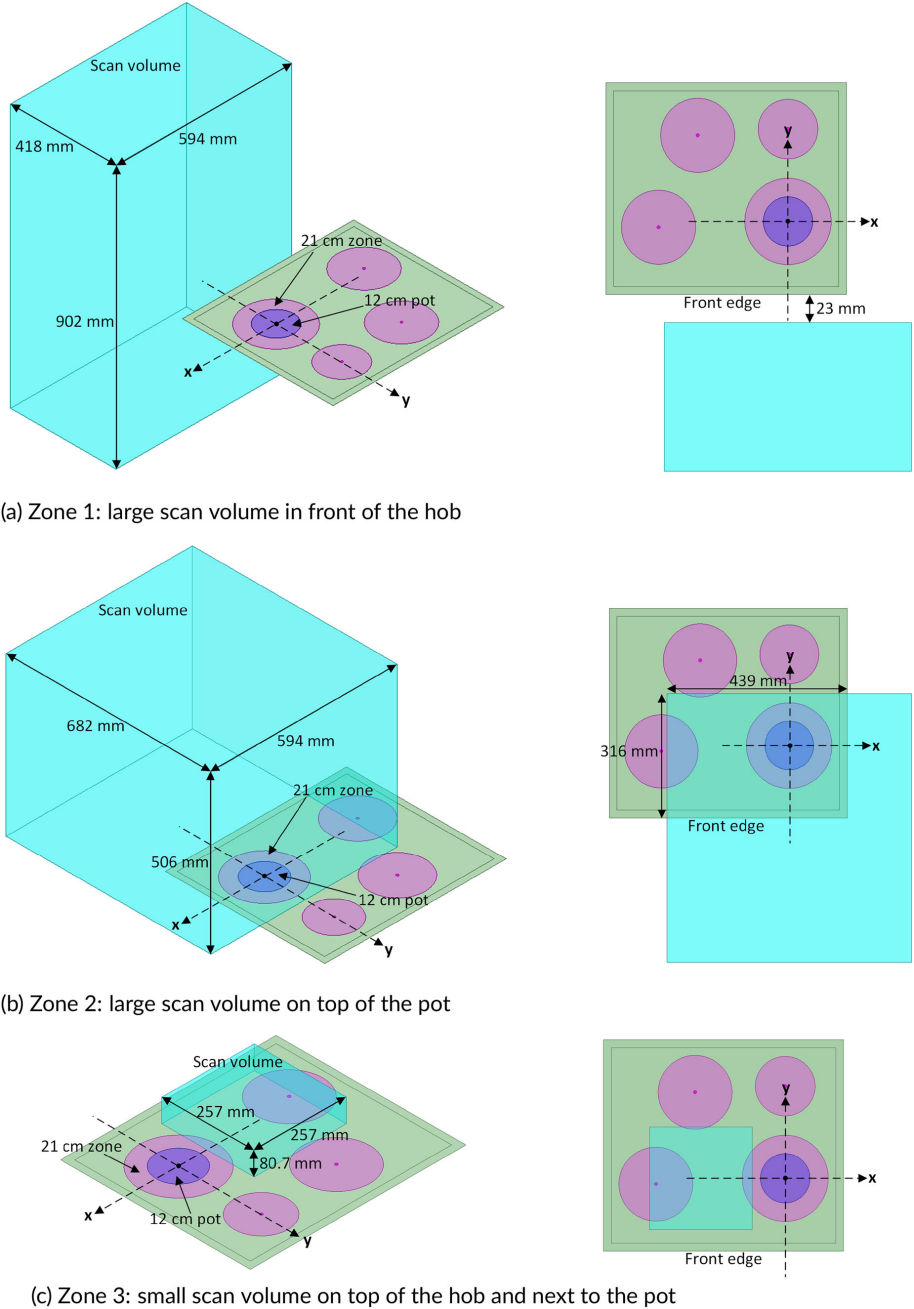
Volume scan setup for the three zones with device A as an example; left: perspective views; right: top views. The top surface of the hob lies at the plane z=0. The origin of the coordinate system is at the center of the target heating zone for devices A and B or at the middle point of the front edge for devices B and D. +x and +y point to the right and the back of the hob, respectively, when the user/bystander is standing in the front and facing the hob. (a) Zone 1: large scan volume in front of the hob; (b) Zone 2: large scan volume on top of the pot; (c) Zone 3: small scan volume on top of the hob and next to the pot.

For the maximum exposure configurations identified in Section 3, high‐resolution volume scans of the 3D distribution of the incident *H*‐field were performed with the DASY6 Module WPT V2.0 with the objectives to obtain comprehensive data about the incident field, which can be used for compliance evaluation with the RL and also can be used as a field source or to derive a surrogate coil model for dosimetric simulations. Three subspaces were measured to reduce the total measurement time:

- Zone 1 (Figure 2a): 594 mm long × 418 mm wide × 902 mm high with a grid resolution of Δ = 22 mm covering the space in front of the hob and aligned with the center of the heating zone (for fixed‐zone hobs) or the pot (for flex‐zone hobs), with the closest measurement plane 23 mm—that is, the minimum clearance defined in the installation instructions of the four sample hobs—away from the front edge of the hob. The volume is sufficient for a dosimetric analysis of persons standing in front of the hob to be performed.
- Zone 2 (Figure 2b): 594 mm long × 682 mm wide × 506 mm high with a grid resolution of Δ = 22 mm covering the space above the pot and aligned with the center of the heating zone (for fixed‐zone hobs) or the pot (for flex‐zone hobs). The volume overlaps sufficiently with the heating zone under test, with the lowest measurement plane 79 mm above the hob surface to avoid collision with the pot and the cooling unit. The volume is designed for a dosimetric analysis of a person bending over the hob and/or of the person's forearm approaching the pot to be performed.
- Zone 3 (Figure 2c): 257 mm long × 257 mm wide × 80.7 mm high with a grid resolution of Δ = 7.33 mm covering the space directly above the hob and aligned with the center of the heating zone (for fixed‐zone hobs) or the pot (for flex‐zone hobs); the lowest measurement planes are 8–12 mm above the hob surface. The volume is designed to allow a dosimetric analysis of a person's hand next to the pot.

During the field measurements, the water in the pot was continuously cooled by a system consisting of a cooling unit fixed within the water and a chiller to keep the water temperature below the boiling point; the power level was reduced to maintain continuous heating. The field was scaled to the maximum power level afterwards for compliance evaluation.

### 4.2 Results

**Figure 3:**
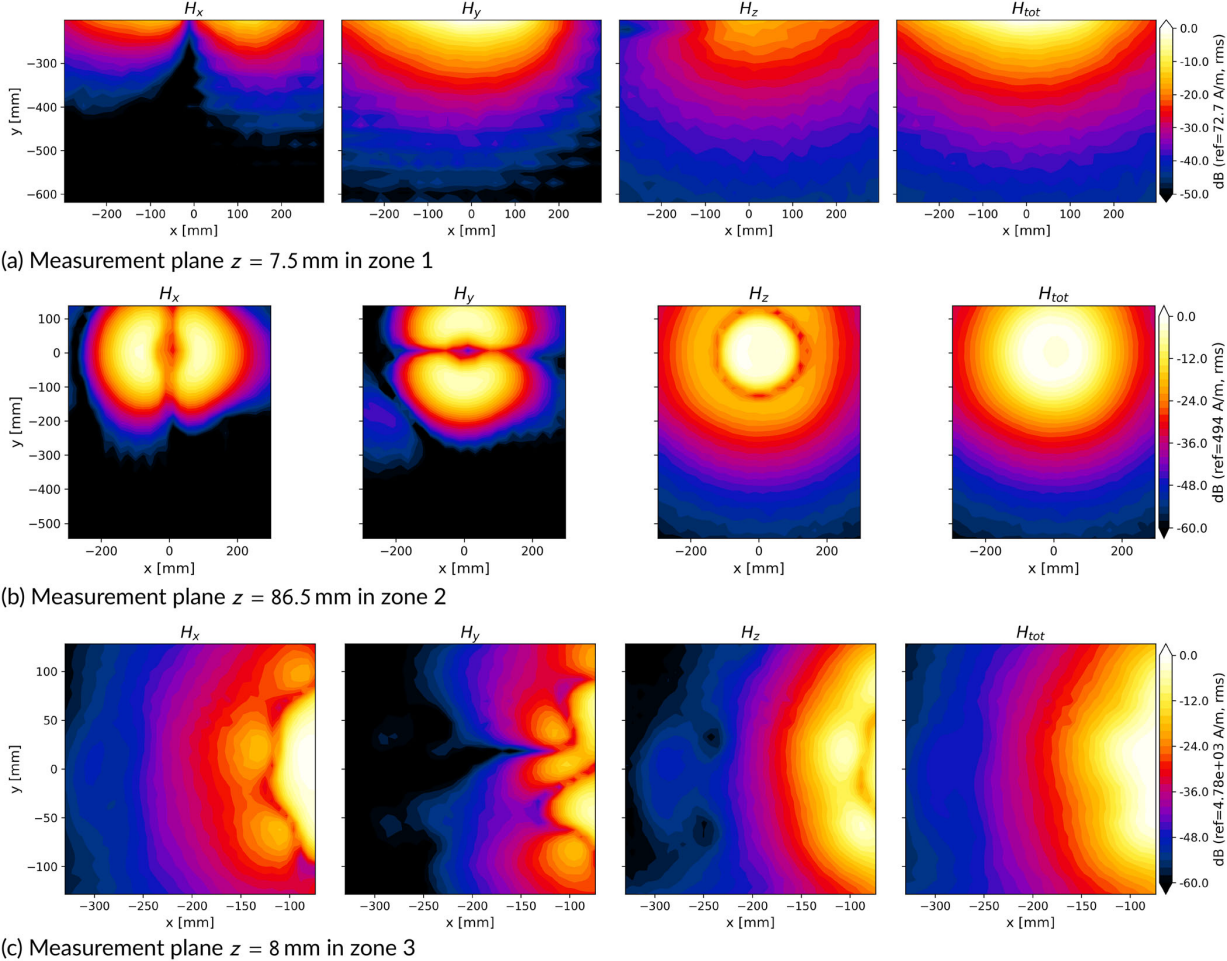
Distribution of the *H*‐fields at different horizontal measurement planes for device A under conditions of single‐pot operation. The rms values of the *H*‐fields at each measurement plane have been normalized to the maximum total *H*‐field at that plane (displayed next to the color bars, corresponding to the worst‐case power setting) and then converted to dB values. z=0 mm corresponds to the hob surface. (a) Measurement plane z=7.5 mm in zone 1; (b) Measurement plane z=86.5 mm in zone 2; (c) Measurement plane z=8 mm in zone 3.

The fundamental frequencies were 31.9, 23.7, 28.9, and 45.0 kHz for devices A–D, respectively. The *H*‐fields at the harmonic frequencies were found to be at least 26 dB lower than that at the fundamental frequency, and thus the impact of the harmonics is negligible. The *H*‐field distributions at the horizontal measurement planes extracted from the three volume scans for device A are presented in Figure 3, showing the field decay when moving away from the hob (Figure 3a), patterns for the spiral heating coil (Figure 3b), and the high fields close to the active coil (Figure 3c). The uncertainty of the total *H*‐field measurement was estimated to be 1.1 dB (k=2) (Schmid & Partner Engineering AG 2023).

**Figure 4:**
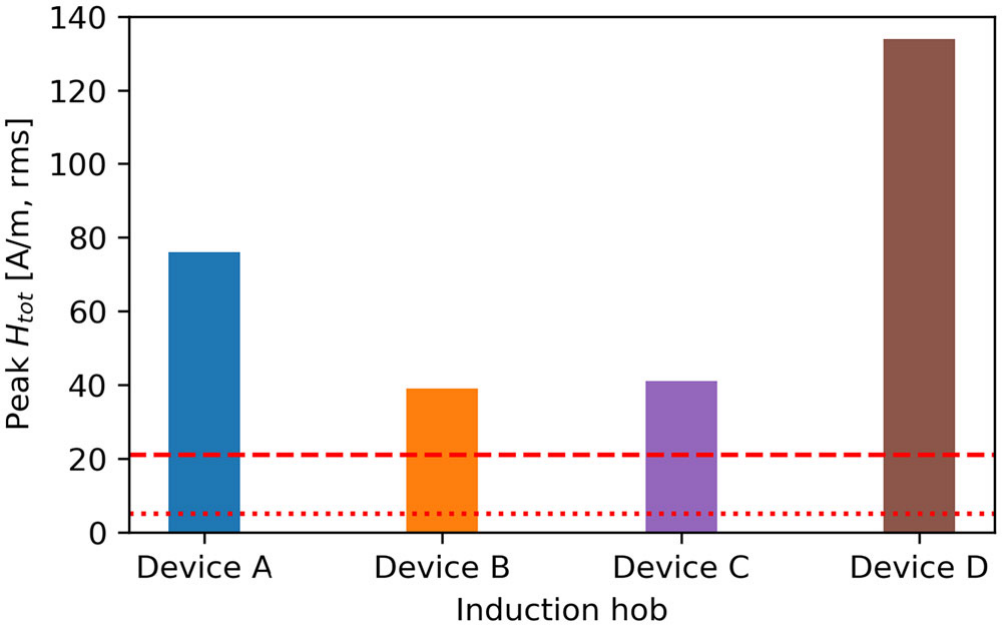
The maximum total *H*‐field measured in front of the hobs at a distance corresponding to the minimum clearance specified in the installation instructions of the hobs. The red dashed line indicates the RL specified in ICNIRP 2010 (21 A/m, rms), and the red dotted line indicates the RL specified in ICNIRP 1998 (5 A/m, rms). *H*‐field strengths have been scaled to the worst‐case power setting.

**Figure 5:**
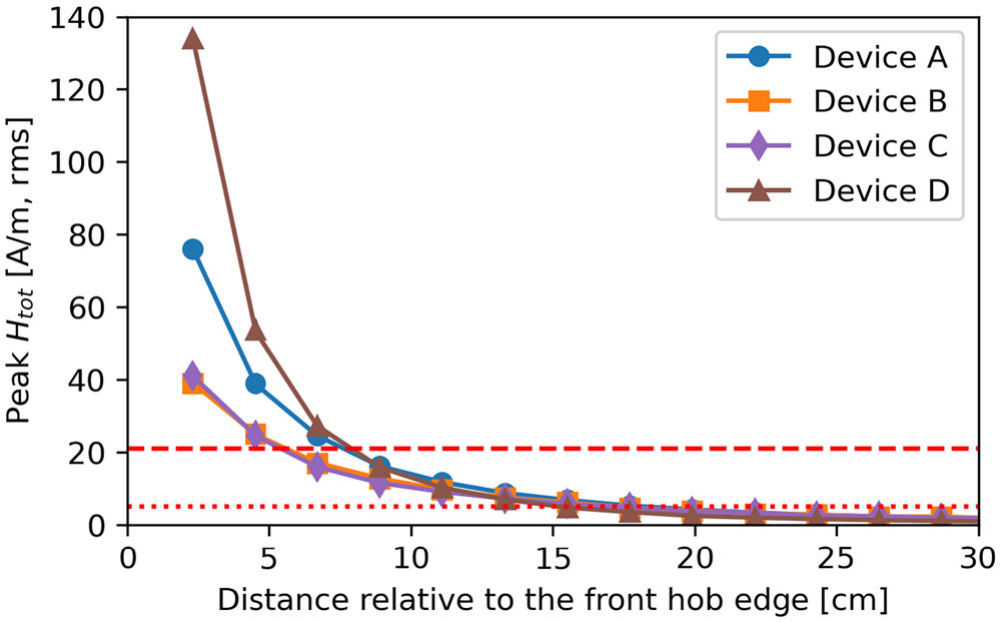
The maximum total *H*‐field measured at different distances (y‐direction) relative to the front edge of each hob. The red dashed line indicates the RL specified in ICNIRP 2010 (21 A/m, rms), and the dotted line indicates the RL specified in ICNIRP 1998 (5 A/m, rms). *H*‐field strengths have been scaled to the worst‐case power setting.

**Figure 6:**
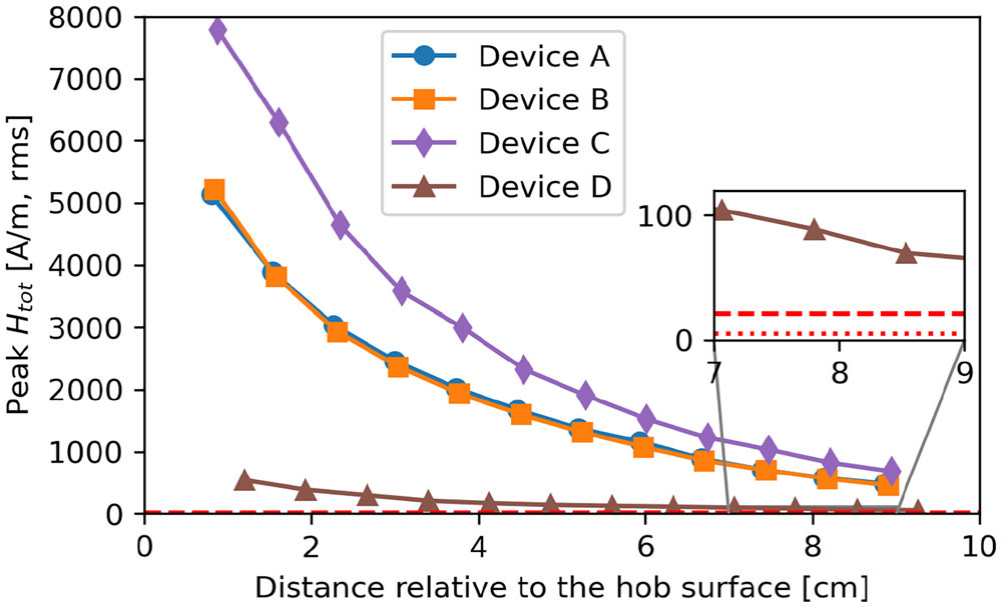
The maximum total *H*‐field measured at different distances (z‐direction) relative to the top surface of each hob. The red dashed line indicates the RL specified in ICNIRP 2010 (21 A/m, rms), and the red dotted line indicates the RL specified in ICNIRP 1998 (5 A/m, rms). *H*‐field strengths have been scaled to the worst‐case power setting.

Figure 4 shows the maximum total *H*‐field measured in front of the hobs (i.e., zone 1) without interpolation or extrapolation, at the distance corresponding to the minimum clearance defined by the hob manufacturers (i.e., 23 mm and 0 mm from the front edges of the hob and the kitchen furniture, respectively). Figure 5 describes the *H*‐field measured in the front as a function of distance relative to the front hob edge. All of the tested devices exceed the RL limits specified in ICNIRP 1998 and ICNIRP 2010 at a position next to the hob, but all of them meet the limits when measured as described in IEC 62233 (IEC International Electrotechnical Commission 2005), that is, at a distance of 30 cm from the front edge. For the evaluated cases, the *H*‐field in front of the hobs is up to 180 times (45.1 dB) higher than at 30 cm. These findings, which are in line with those of (Christ et al. 2012; Gryz et al. 2020), demonstrate that the product standard IEC 62233 does not guarantee safety and should be revised. Figure 6 shows the *H*‐field measured next to the pot as a function of distance relative to the hob surface. Obviously, all of the tested devices exceed the RL limits.

## 5 Determination of the Maximum Contact Currents

### 5.1 Methods

**Figure 7:**
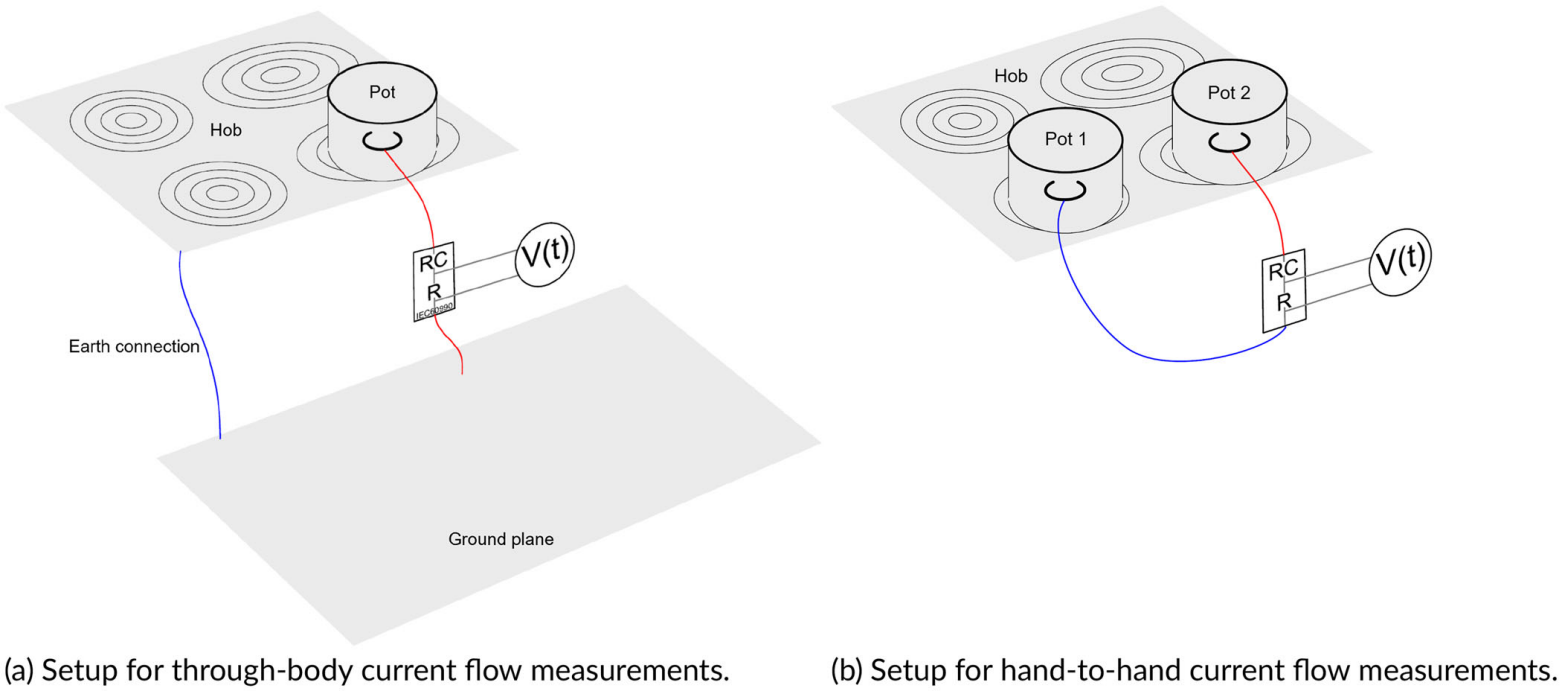
Contact current measurement setups; current flow was recorded in the time domain with a differential voltage probe connected to an optically isolated oscilloscope. (a) Setup for through‐body current flow measurements, with the current flowing through the IEC 60990 body impedance simulating circuit; the hob was mounted on a frame at a height of 70 cm from the floor; a ground plane was positioned in front of the hob and was connected to the shield of the hob. (b) Setup for hand‐to‐hand current flow measurements, with the current flowing between two pots through the hand‐to‐hand impedance‐simulating circuit and the hob mounted at a height of 70 cm. (a) Setup for through‐body current flow measurements. (b) Setup for hand‐to‐hand current flow measurements.

**Figure 8:**
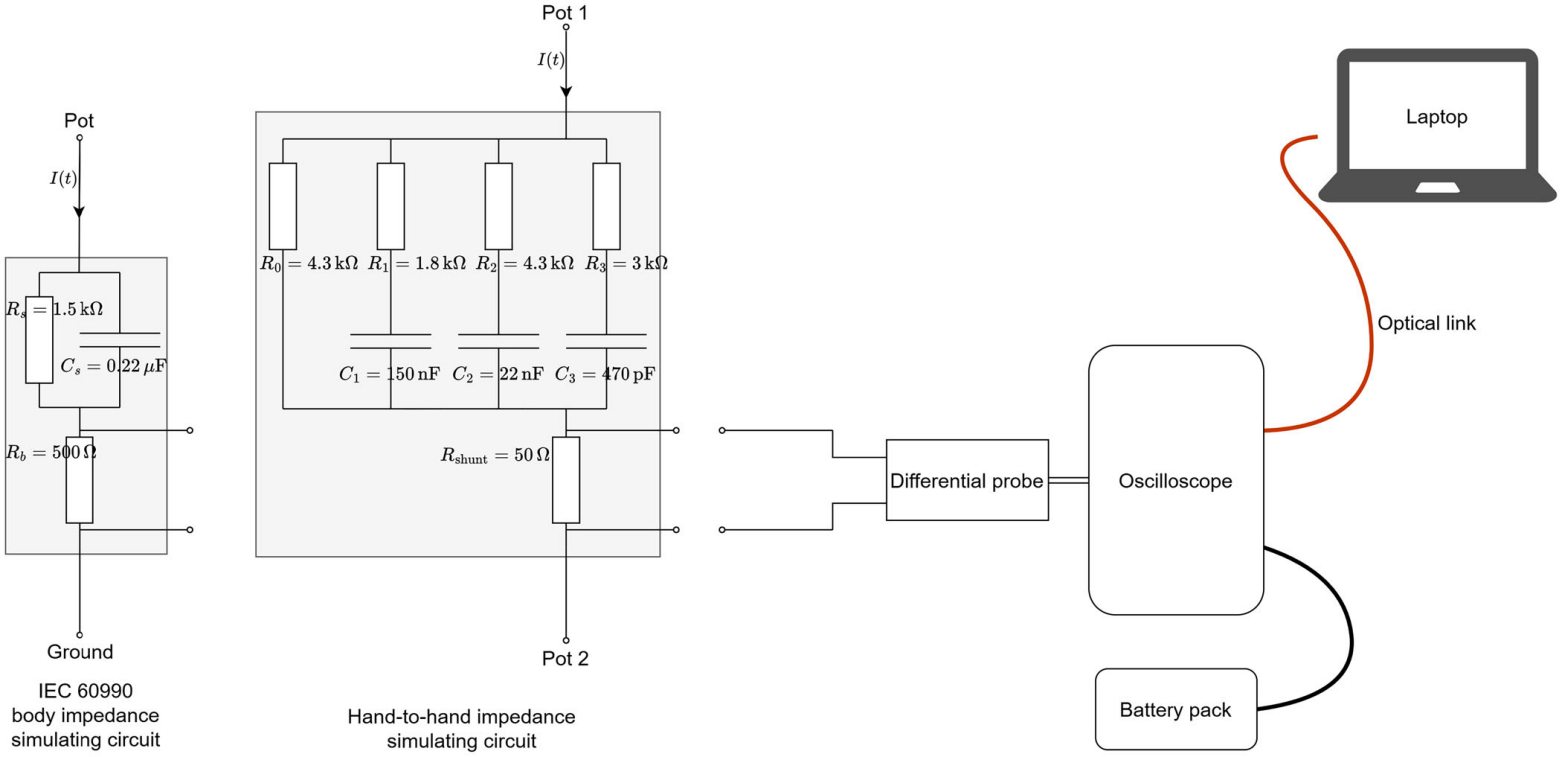
The components of the contact current measurement system, from left to right: the body impedance simulating circuits; a differential voltage probe (R&S RT‐ZD003); a battery‐powered optically isolated oscilloscope (PicoScope 5444D); and a laptop for measurement control and data acquisition.

Figure 7a shows the setup for measurement of the contact currents, in which a user touching one pot with current flowing through the body to a ground plane is simulated. Figure 7b depicts a setup in which the user touches two pots and has current flowing hand‐to‐hand. A traceable alternating current (AC) voltmeter (Keithley 2001) with a 50 Ω shunt resistor was used to calibrate the contact current measurement system (Figure 8).

Impedance‐simulating circuits, which have lower measurement uncertainty than direct human touch or limb current measurements (Alanko et al. 2011), were used to assess the contact currents. The body impedance circuit from (IEC International Electrotechnical Commission 2016) was used to emulate current flowing to the ground, aligning within <3 dB of the 10th0th percentile lowest human body impedance from 10 kHz to 1 MHz (Kamimura et al. 2018). The hand‐to‐hand impedance model derived, based on (Chinen et al. 2015), approximates measurement results of (Chinen et al. 2015) within 5% from 1 kHz to 1 MHz.

Pots of three dimensions—12 cm diameter × 7 cm high; 16 cm diameter × 10 cm high; and 28 cm diameter ×× 20 cm high—were selected to assess contact current flow to the ground. Two pairs of pots—12 cm diameter ×× 7 cm high and 28 cm diameter × 20 cm high; 22 cm diameter × 12 cm high and 22 cm diameter × 14 cm high—were selected to assess the hand‐to‐hand contact current flow. A larger pot is expected to couple more radiofrequency (RF) energy from the heating elements; however, if the pot extends beyond the element, it might capacitively short the RF energy to the hob's shielding. For fixed‐zone hobs, the pots were centered over the largest or bridged heating element. For flex‐zone hobs, the pots were placed on the positioning marks and then offset to activate multiple elements while recording. Contact currents were recorded continuously, with the maximum extracted during post‐processing. All hobs were tested at the maximum power level.

The post‐processing was performed in the following steps. First, the raw signal I(t) captured was transformed to the frequency domain I(f). Then, a frequency domain filter, representing the inverse of the RL slope defined in (ICNIRP 2010) and normalized to the limit at 100 kHz was applied to I(f) to obtain the weighted frequency domain data $I_{\text{weighted}}\left(f\right)$. The weighting factors implemented were based on the recommendations of (ICNIRP 2003). A high‐pass filter ($f_{c}<10$ kHz) was applied to $I_{\text{weighted}}\left(f\right)$ to suppress low‐frequency noise, followed by the transformation of $I_{\text{weighted}}\left(f\right)$ back to the time domain $I_{\text{weighted}}\left(t\right)$. Finally, the absolute peak value $I_{\max}$ was extracted from $I_{\text{weighted}}\left(t\right)$ and compared with the limit at 100 kHz, that is, 28.3 mA (peak) (ICNIRP 2010).

The same contact current RL limits were defined in (ICNIRP 1998) and (ICNIRP 2010) for frequencies of up to 10 MHz. In the 2.5–100 kHz range, the RL is $0.2f_{\text{kHz}}$ mA (rms). Neither guideline specifies whether the limits apply to rms or peak current values; our assumption, based on (Alanko et al. 2011), is that the RL limits refer to rms values. Since the guidelines do not define an averaging duration for contact current limits, and induction hobs generate complex waveforms, we multiply the rms limit by $\sqrt{2}$ to convert it to a peak limit for direct comparison with instantaneous peak currents.

### 5.2 Results

The measurement results of the maximum contact currents, shown in Table 3, have an estimated uncertainty of 0.84 dB (k=2).

**Table 3:** Maximum values of contact currents (in mA) flowing through the body to the ground when cooking with a single pot (1, 2, or 3), or from one hand to the other when cooking with a pair of pots (1 or 2); the values are compared to a peak value for the limit of 28.3 mA.

	Through‐body current flow to the ground			Hand‐to‐hand current flow	
				Pair 1	Pair 2
	Pot 1	Pot 2	Pot 3	Ø12 cm×H 7 cm	Ø22 cm×H 12 cm
Device	Ø12 cm×H 7 cm	Ø16 cm×H 10 cm	Ø28 cm×H 20 cm	Ø28 cm×H 20 cm	Ø22 cm×H 14 cm
A	22.9	21.5	18.0	32.5	42.3
B	25.9	29.4	19.1	42.4	32.5
C	13.5	22.9	20.8	25.0	20.5
D	12.9	18.4	28.4	20.8	35.7

For the contact current flowing through the body to the ground, the maximum contact currents measured with the body impedance simulating circuit were generally within the RL range for all induction hobs tested. Notably, the highest contact currents (observed on device B), which occur during very rapid (≈1 μs) transients, are likely due to switching events in the heating element driver circuits and exceed the RL by only 0.34 dB. These transient signals are capacitively coupled to the pots. The results suggest that the body contact current depends on a balance between capacitive coupling from the heating element to the pots and its coupling to the hob's shield.

For hand‐to‐hand contact current—where each hand is touching a different pot— generally higher currents were measured. Except for device C, all hobs exceed the RL, with devices A and D exceeding it by up to 3.5 dB. No clear correlation was found between contact current flow and pot size or hob type. As for through‐body current flow, currents exceeding the RL were observed only during μs‐range transient events. Depending on synchronous or asynchronous operation, the potential differences between pots may constructively superimpose, leading to higher contact currents.

## 6 Evaluation of the Induced Fields and BR Quantities

### 6.1 Methods

**Figure 9:**
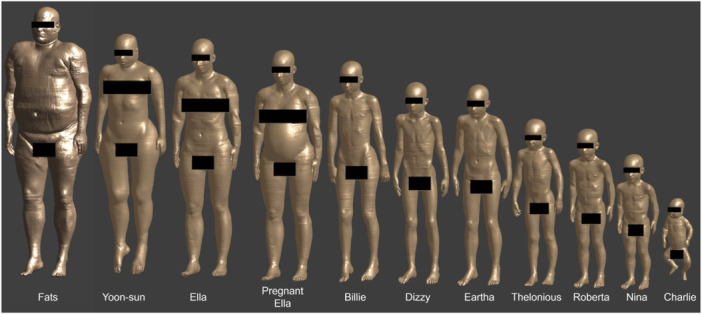
ViP models used in the dosimetric simulations.

Since the incident fields are not compliant with the RL limits, the next step was to compare the induced fields with the BR. The simulation platform Sim4Life V7.2 (ZMT Zurich MedTech AG) with the Virtual Population (ViP) anatomical models posed in a variety of foreseeable exposure scenarios was used to determine the induced fields. The ViP models used in this study (except the fetuses) are shown in Figure 9, and detailed information about the models is summarized in Table 4 (Gosselin et al. 2014). The finding of Christ et al. (2012) regarding the worst‐case human models guided the selection of the anatomical models. The tissue properties were configured according to the IT'IS tissue database (IT'IS Foundation n.d). The ViP models were discretized at resolutions as fine as 0.7 mm. The peak values of the induced field quantities 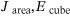, and 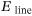 were extracted from the dosimetric simulation results.

**Table 4:** Information about the ViP models used in the dosimetric simulations.

Name	Gender	Age	Height	Weight	BMI	Tissue number	Version
			[m]	[kg]	[${\;\text{kg/m}\;}^{2}$]		
Fetus 1	N.A.	3 months	N.A.	0.015	N.A.	15	1.1
Fetus 2	N.A.	7 months	N.A.	1.4	N.A.	19	1.1
Fetus 3	Female	9 months	N.A.	2.7	N.A.	26	1.1
Charlie	Female	8 weeks	N.A.	4.3	N.A.	61	1.1
Nina	Female	3 years	0.92	13.9	16.4	104	1.1
Roberta	Female	5 years	1.09	17.8	14.9	302	3.1
Thelonious	Male	6 years	1.16	18.6	13.8	299	3.1
Eartha	Female	8 years	1.36	29.9	16.2	306	3.1
Dizzy	Male	8 years	1.37	25.3	13.5	306	3.1
Billie	Female	11 years	1.49	34.0	15.3	305	3.1
Ella	Female	26 years	1.63	57.3	21.6	305	3.1
Pregnant Ella	Female	26 years	1.63	58.7	22.0	76	1.3
Yoon‐sun	Female	26 years	1.52	54.6	23.6	322	3.1
Fats	Male	37 years	1.82	119	36.0	305	3.2

**Figure 10:**
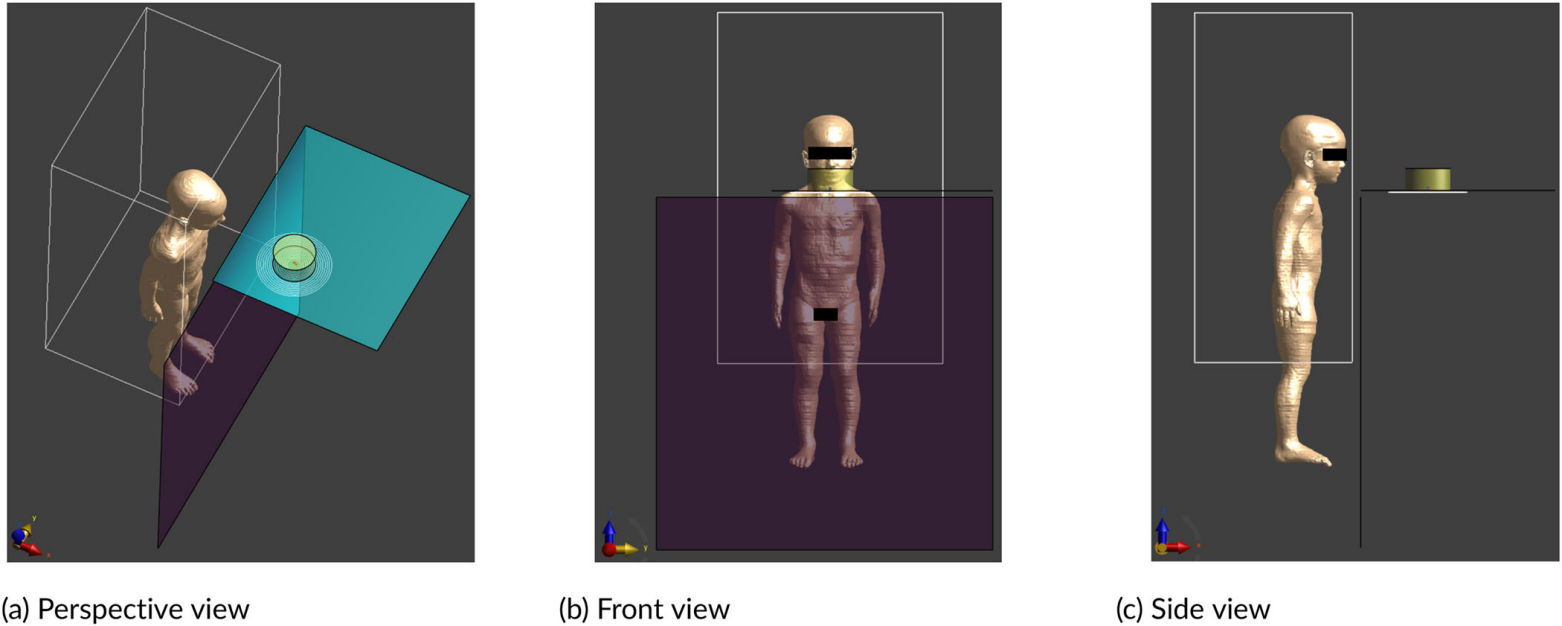
Simulation model for Nina standing in front of device A. The front scan volume (for zone 1) and the induction hob model are also shown. (a) Perspective view; (b) Front view; (c) Side view.

**Figure 11:**
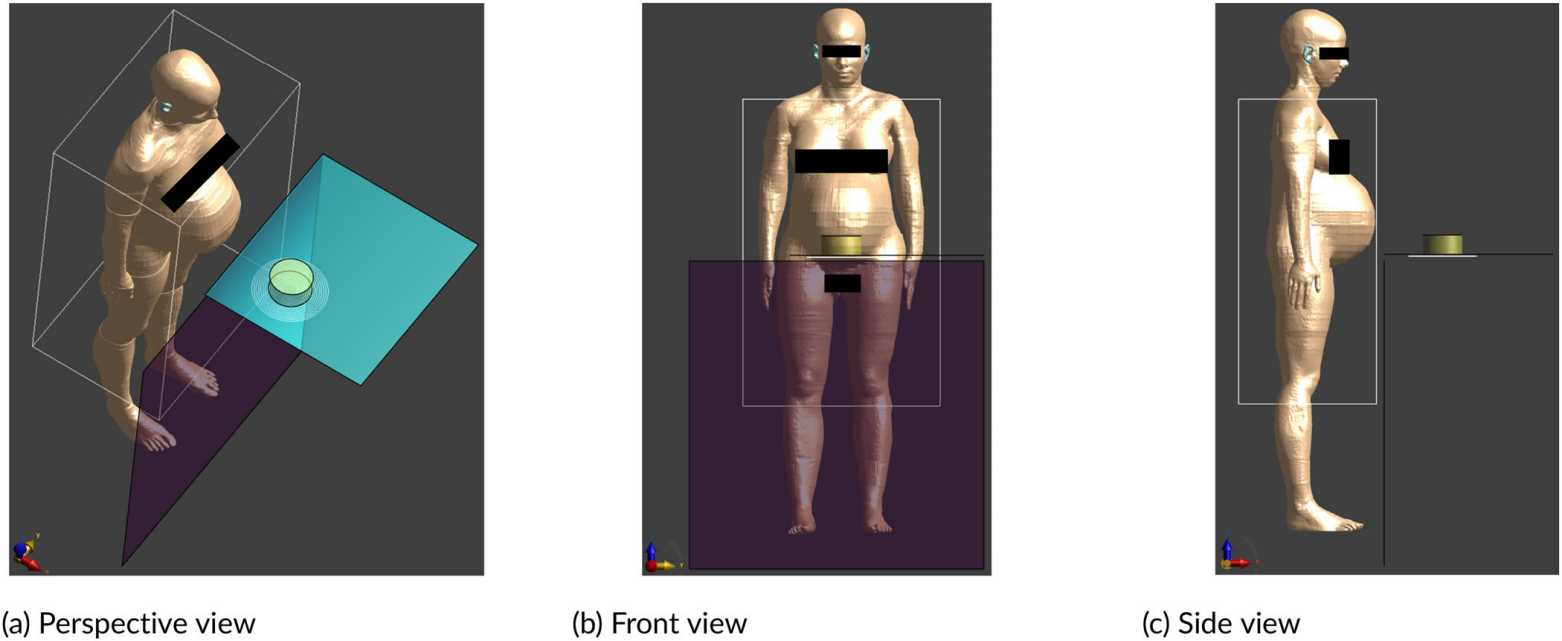
Simulation model for 9‐month‐pregnant Ella standing in front of device A. The front scan volume (for zone 1) and the induction hob model are also shown. (a) Perspective view; (b) Front view; (c) Side view.

**Figure 12:**
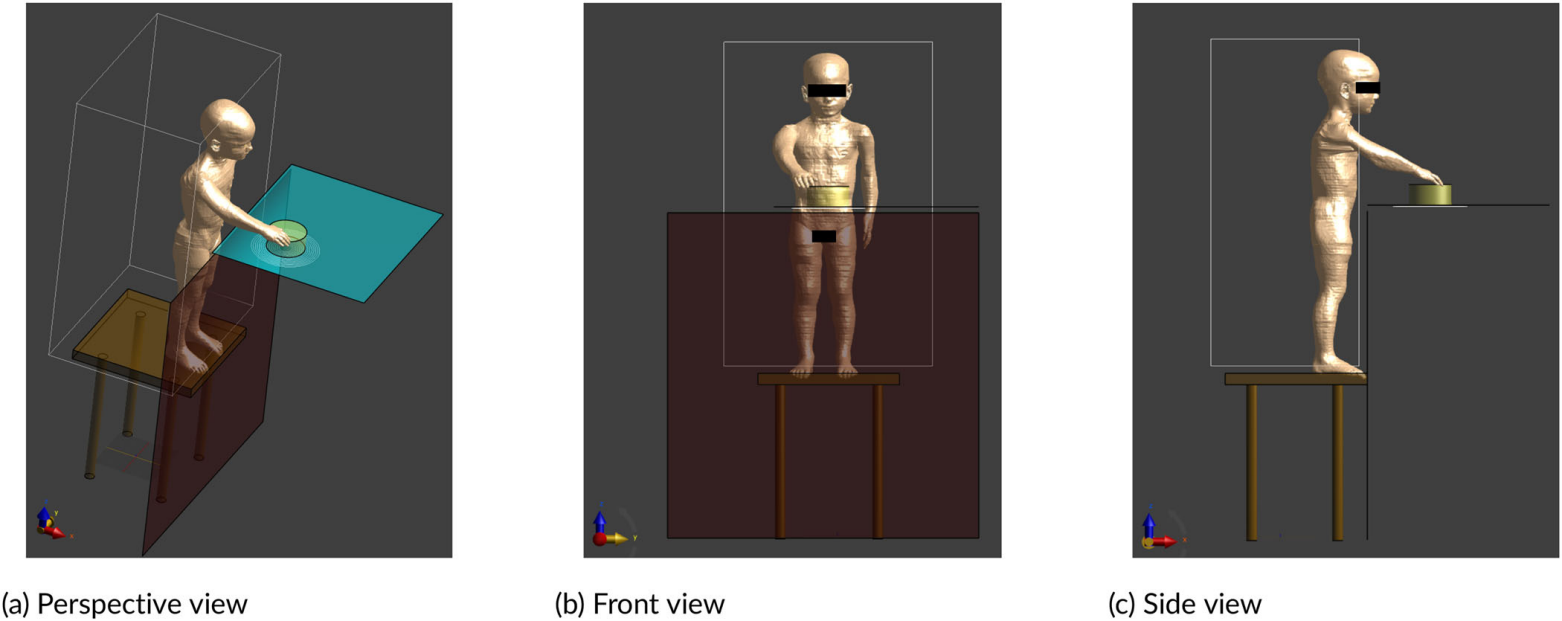
Simulation model for special case 1, whereby Nina stands on a chair in front of device A to stir a pot. The front scan volume (for zone 1) and the induction hob model are also shown. (a) Perspective view; (b) Front view; (c) Side view.

**Figure 13:**
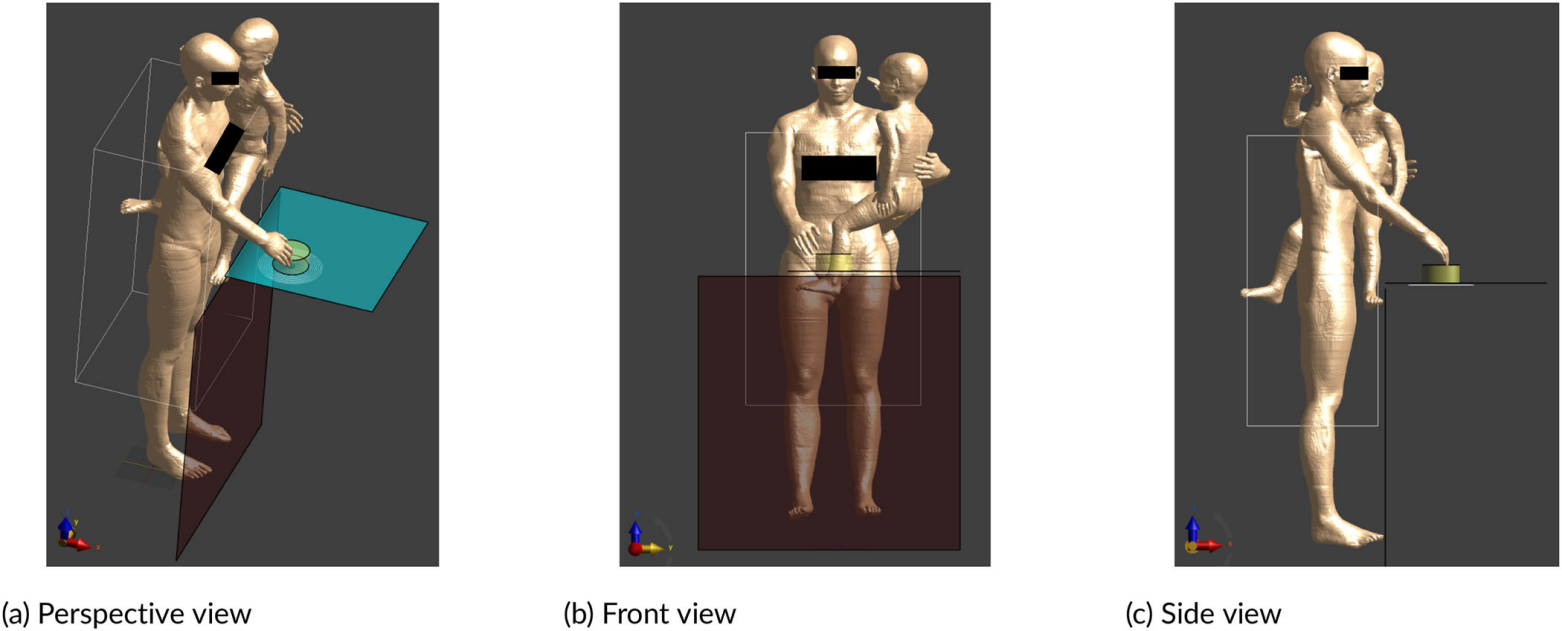
Simulation model for special case 2, whereby Ella holds Nina on her hip and cooks with one arm on device A. The front scan volume (for zone 1) and the induction hob model are also shown. (a) Perspective view; (b) Front view; (c) Side view.

**Figure 14:**
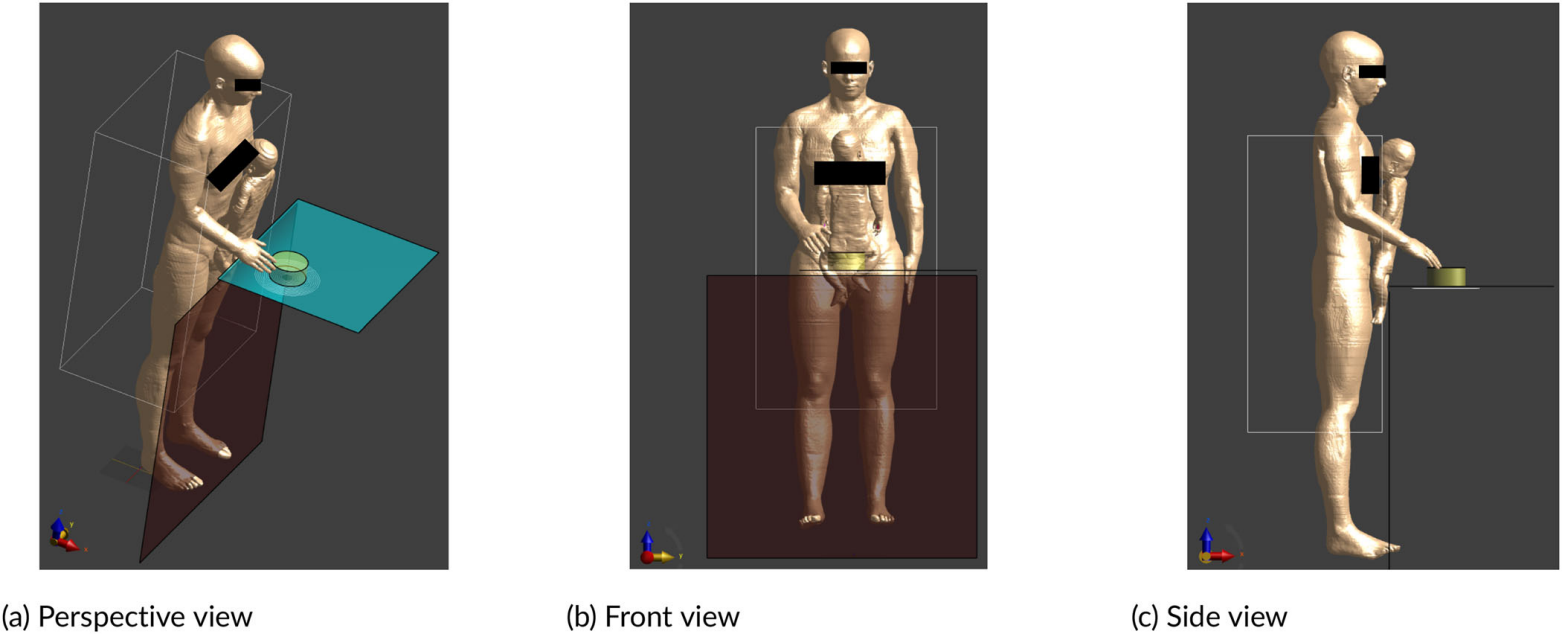
Simulation model for special case 3, whereby Ella holds Charlie on her chest and cooks with one arm on device A. The front scan volume (for zone 1) and the induction hob model are also shown. (a) Perspective view; (b) Front view; (c) Side view.

The exposure scenario whereby the user/bystander is positioned in front of the hob was studied with 10 ViP models of different ages, including the three versions of the pregnant Ella model with 3‐month‐old, 7‐month‐old, and 9‐month‐old fetuses. In the simulation of this scenario, the measured 3D *H*‐fields in zone 1 were used as the source. Each ViP model was placed in the volume corresponding to zone 1. Along the x‐axis, each model was centered in the volume. Along the y‐axis, the separation between each model and the front edge of each hob was adjusted according to the minimum clearance requirements from the installation instructions of the hobs. Along the z‐axis, each adult model was positioned so as to stand at a virtual ground plane located 85 cm below the hob surface. The child ViP models were lifted to bring their heads to the height of the hob surface. The simulation models for Nina and pregnant Ella (9 months) are shown in Figures 10 and 11, respectively. The ViP models used in the dosimetric simulations with the measured *H*‐fields as the source were truncated at the boundary of the scan volumes. Zone 1 covers *H*‐fields with a field decay (relative to the maximum *H*‐field in the zone) of > 20 dB and includes all tissues around the peak induced fields. The error due to the truncation of the ViP models has been determined to be less than 0.2 dB. An active cooking scenario, whereby the user is stirring a pot, was studied with the following three special cases:

- Special case 1: 3‐year‐old child Nina stands on a chair in front of the hob to stir a pot (Figure 12),
- Special case 2: adult Ella carries her 3‐year‐old child Nina on her hip and cooks with one arm (Figure 13),
- Special case 3: adult Ella carries her 8‐week‐old child Charlie on her chest and cooks with one arm (Figure 14).

**Figure 15:**
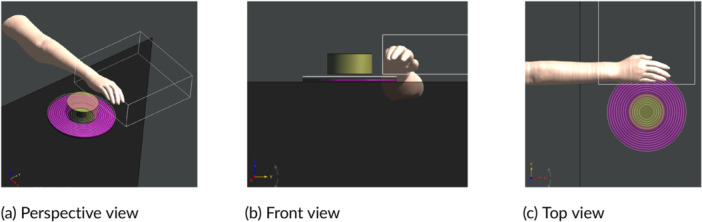
Simulation model for Yoon‐sun's right hand positioned above device A close to the pot. The top scan volume (for zone 3) and the induction hob model are also shown. (a) Perspective view; (b) Front view; (c) Top view.

Since the measured volumes are not sufficient to include these special ViP model configurations, simulation models of the specific hobs were created and validated with the field distribution measured. The simulation models consist of coils implemented in terms of concentric loops, shields beneath the coils, and a pot‐mimicking cylinder on top of the coils. The outer diameters of the surrogate coils were set according to the diameters of the heating zones for fixed‐zone hobs or those of the heating coils for flex‐zone hobs. The model geometries and coil currents were optimized to match the measured 3D *H*‐field distributions, resulting in deviations of ≤2 dB. The hand exposure scenario was studied with Yoon‐sun's right hand placed directly above the hob and next to the pot, as shown in Figure 15. Yoon‐sun was selected because it was created based on high‐resolution cryosection images, offering greater anatomical detail—especially for nerves and vessels—compared to ViP models derived from MRI data. The measured *H*‐field in zone 3 was used as the source in the hand exposure simulation. The volume of zone 3 is sufficiently large such that the uncertainty due to the truncation of the hand model is less than 0.2 dB.

### 6.2 Results

**Figure 16:**
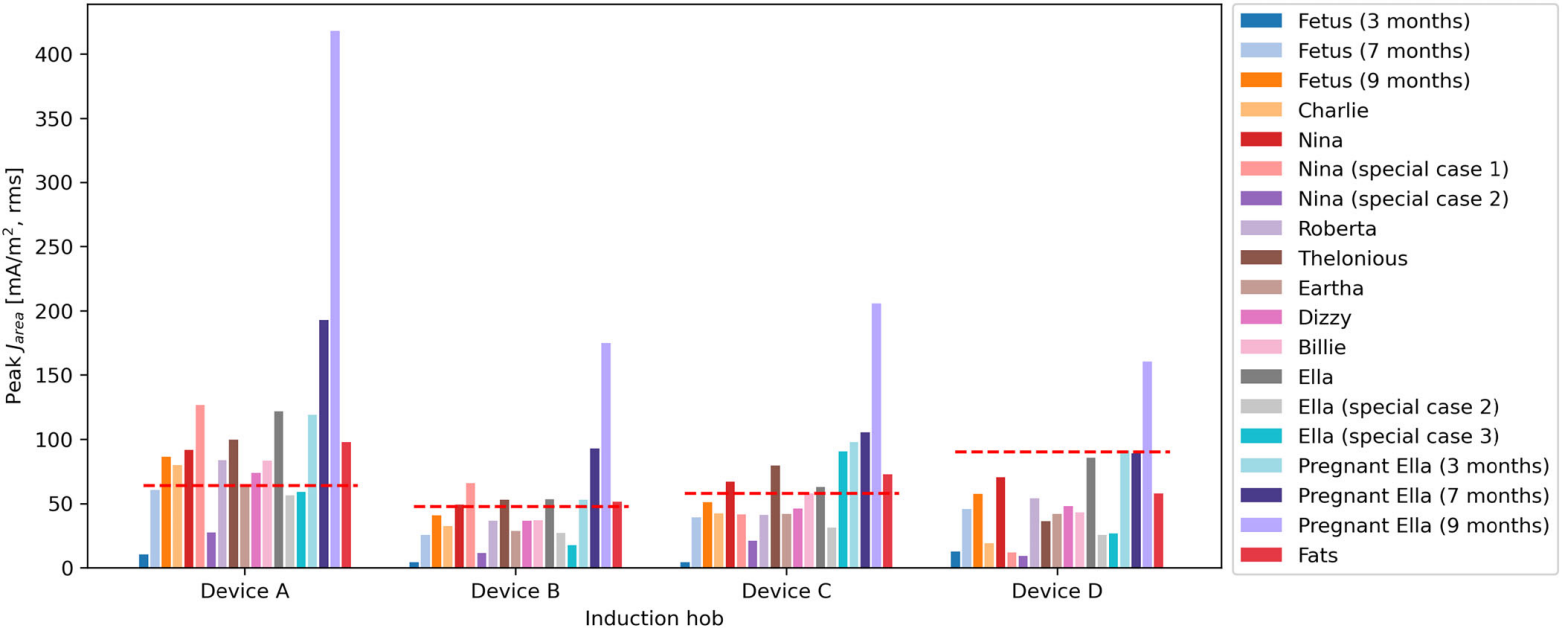
The maximum 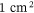 area averaged induced current density 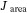 simulated at distances corresponding to the minimum clearances specified in the installation instructions of the hobs. The different bars for each hob represent results for different ViP models (hand exposure excluded). The red dashed lines show the BR limits for the different hobs. Peak 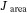 levels in the amniotic fluid are excluded in this chart.

**Figure 17:**
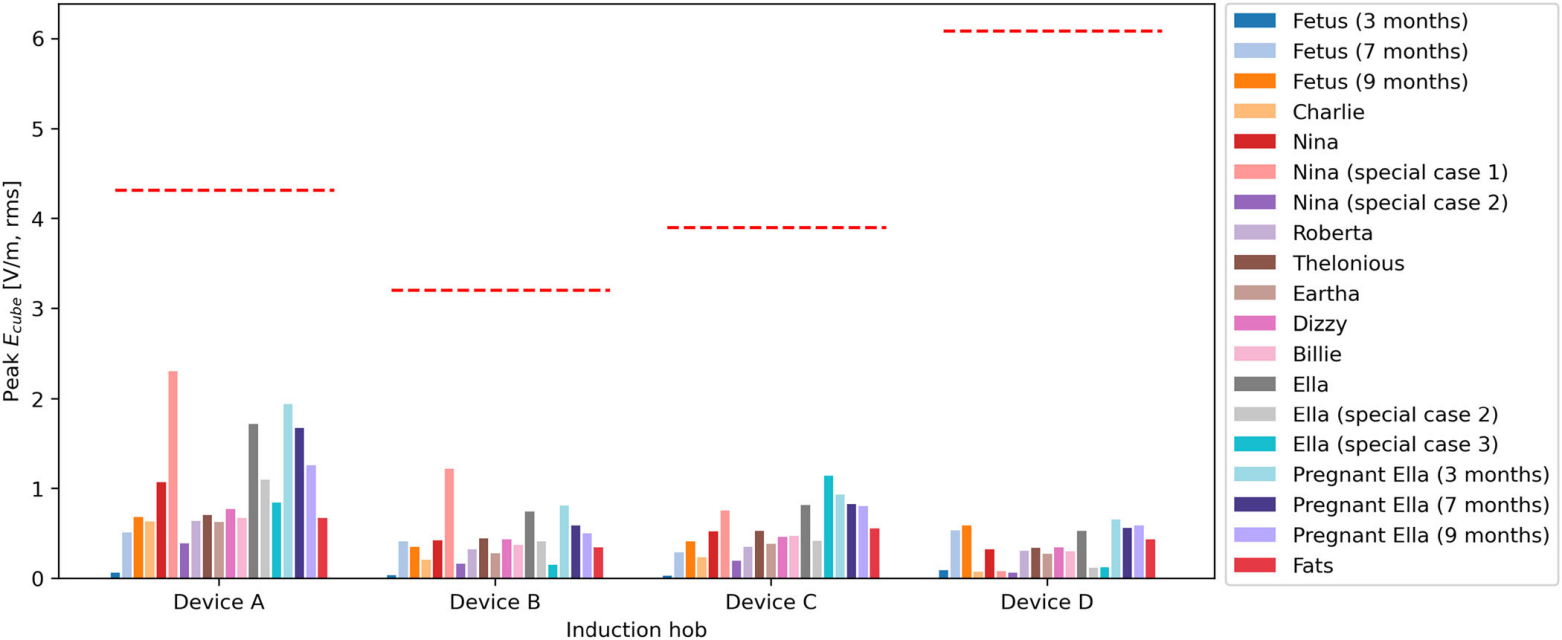
The maximum $8\;{\;\text{mm}\;}^{3}$ cube averaged induced *E*‐field ${\;\text{E}\;}_{\text{cube}}$ simulated at distances corresponding to the minimum clearances specified in the installation instructions of the hobs. The different bars for each hob represent results for different ViP models (hand exposure excluded). The red dashed lines show the BR limits for the different hobs.

**Figure 18:**
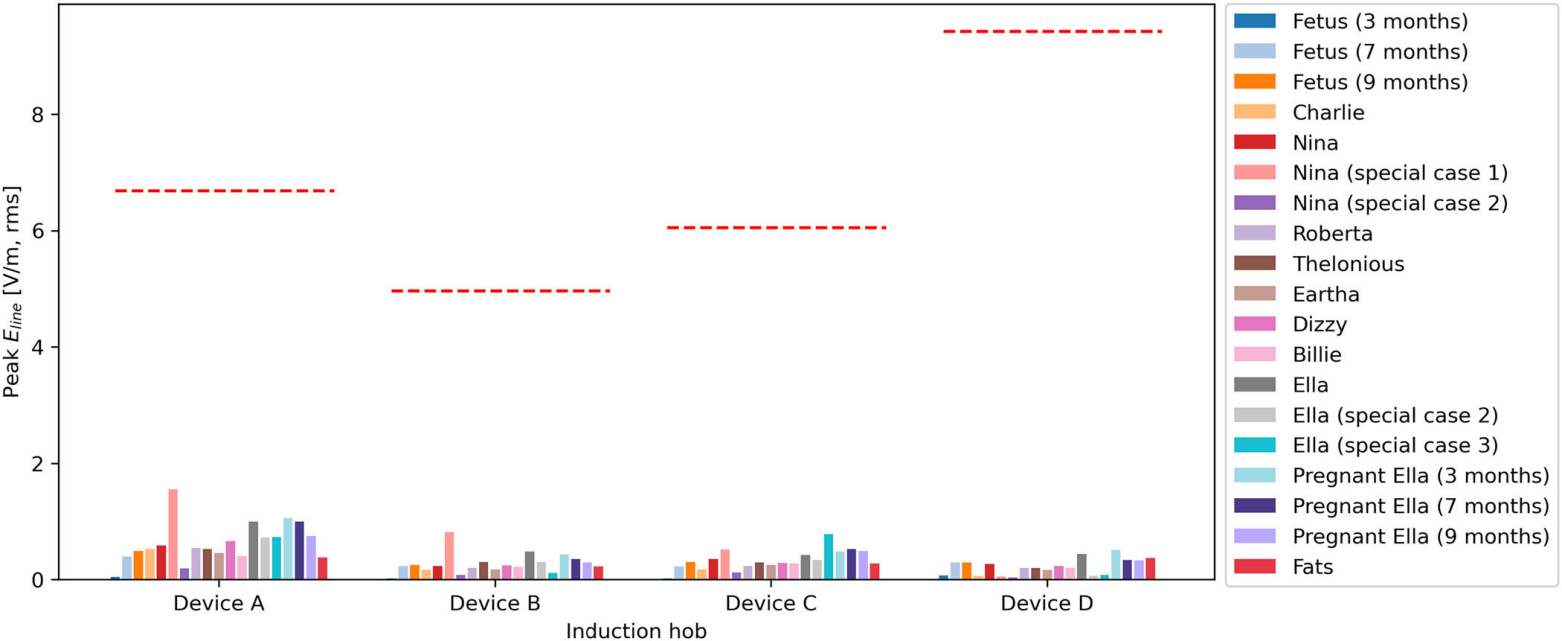
The maximum 5 mm line averaged induced *E*‐field ${\;\text{E}\;}_{\text{line}}$ simulated at distances corresponding to the minimum clearances specified in the installation instructions of the hobs. The different bars for each hob represent results for different ViP models (hand exposure excluded). The red dashed lines show the BR limits for the different hobs.

The peak induced fields are provided in Figures 16, 17, 18. They have been scaled to the worst‐case power level. The uncertainty of the simulated peak induced fields is 1.8 dB (k=2), assessed according to IEC 63184 (IEC International Electrotechnical Commission 2021). This value accounts for uncertainties related to both the hob model and the computational method. The uncertainty of the hob model equals that of the *H*‐field measurement, since the hobs were either directly modeled by the measured 3D *H*‐fields or modeled by surrogate coils validated by *H*‐field measurements. The uncertainty of the computational method includes contributions from grid resolution, convergence, computation domain size, quasi‐static approximation, and tissue conductivities. The corresponding BR limits are also marked in the figures. 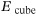 and ${\;\text{E}\;}_{\text{line}}$ results are all lower than the BR limits specified in (ICNIRP 2010) and (IEEE 2019), respectively. However, the maximum 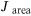 values are higher than the BR in many cases. The induced current densities in the highly conductive amniotic fluid (σ=1.88 S/m) for the 7‐month‐ and 9‐month‐pregnant Ella models have not been taken into consideration in the compliance assessment, as they are irrelevant for nerve excitation risks.

**Figure 19:**
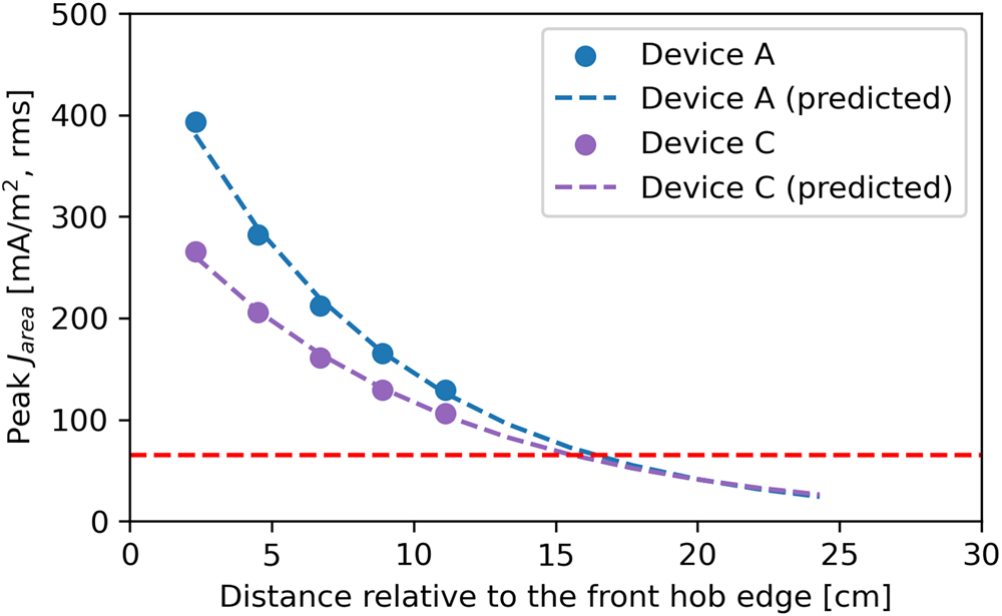
The maximum $1\;{\;\text{cm}\;}^{2}$ area averaged induced current density 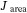 in 9‐month‐pregnant Ella, determined at different distances relative to the front edge of the hob. The red dashed line shows the BR limit for device A; the BR limit for device C (not plotted) is similar. It must be noted that induced current densities in the highly conductive amniotic fluid (σ = 1.88 S/m) are not included, as these are irrelevant to risks due to nerve excitation.

Extra simulations were performed with the 9‐month‐pregnant Ella, which was the worst case found among the full‐body models studied, on devices A and C at larger distances—that is, 67, 89, and 111 mm—from the hob to estimate the compliance distance for 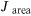, that is, the distance at which the peak 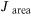 falls below the BR. The results are shown in Figure 19. Based on the exponential curve fitting of data for distances of 23–111 mm, it is predicted that the peak 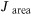, excluding results in the amniotic fluid, becomes compatible with the BR at 17 cm. This finding was confirmed by another simulation on device A at 19 cm distance (not shown in Figure 19). The compliance distance estimated here is consistent with the finding in (Christ et al. 2012), in which a compliance distance between 20 and 25 cm was reported, however, for a coil current producing an *H*‐field of 5 A/m (rms) at a distance of 30 cm.

## 7 Discussion and Conclusions

Different exposure assessment methods and procedures were applied to four different hob appliances (see Tables 5, 6, 7, 8, 9, 10) and evaluated for their suitability in demonstrating compliance with the three exposure safety limits set by ICNIRP (ICNIRP 1998, 2010) and IEEE (IEEE 2019) (see Table 1). In the first column of Tables 5, 6, 7, 8, 9, 10, the maximum *H*‐field measured at 30 cm distance from the hob edge was compared with the RL limits, according to IEC 62233 (IEC International Electrotechnical Commission 2005). In the second column, the maximum *H*‐field measured in zone 1 or zone 3 was compared with the RL limits, according to Tier 2 of IEC 63184 (IEC International Electrotechnical Commission 2021). In the third column, the maximum induced fields were estimated by applying the GGSM method (Liorni et al. 2020) and compared with the BR limits according to Tier 3 of IEC 63184. In the fourth column, corresponding to Tier 4 of IEC 63184, the maximum induced fields of all cases simulated with anatomical models were compared with the BR limits.

**Table 5:** Results of compliance evaluations in terms of the *H*‐field RL (i.e., ) and BR (i.e., ${\;\text{J}\;}_{\text{area‐lim}}$) of ICNIRP 1998 according to different procedures for a user/bystander in zone 1, where is the incident *H*‐field measured. The Tier 3 assessment here is based on the *H*‐field gradient along the y axis (i.e., from the front to the back of the hob).

Device	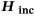 @30 cm/ 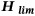	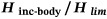	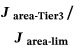	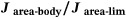
	(IEC 62233)	(IEC 63184 Tier 2)	(IEC 63184 Tier 3)	(IEC 63184 Tier 4)
	[dB] (unitless)	[dB] (unitless)	[dB] (unitless)	[dB] (unitless)
A	−10.4 (0.30)	23.3 (15)	13.0 (4.5)	16.3 (6.5)
B	−9.5 (0.33)	17.9 (7.9)	9.5 (3.0)	11.3 (3.7)
C	−11.9 (0.25)	18.3 (8.2)	9.5 (3.0)	11.0 (3.5)
D	−16.5 (0.15)	28.6 (27)	16.2 (6.5)	5.0 (1.8)

**Table 6:** Results of compliance evaluations in terms of the *H*‐field RL (i.e., ) and ${\;\text{E}\;}_{\text{cube}}$ BR (i.e., ) of ICNIRP 2010 according to different procedures for a user/bystander in zone 1, where is the incident *H*‐field measured. The Tier 3 assessment here is based on the *H*‐field gradient along the y axis (i.e., from the front to the back of the hob).

Device	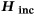 @30 cm/ 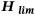	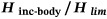	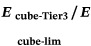	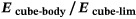
	(IEC 62233)	(IEC 63184 Tier 2)	(IEC 63184 Tier 3)	(IEC 63184 Tier 4)
	[dB] (unitless)	[dB] (unitless)	[dB] (unitless)	[dB] (unitless)
A	−22.9 (7.2 $\times \;10^{-2}$)	10.8 (3.5)	2.0 (1.3)	−5.6 (0.52)
B	−21.9 (8.0 $\times \;10^{-2}$)	5.4 (1.9)	−1.5 (0.84)	−8.4 (0.38)
C	−24.3 (6.1 $\times \;10^{-2}$)	5.8 (1.9)	−1.5 (0.84)	−10.7 (0.29)
D	−29.0 (3.5 $\times \;10^{-2}$)	16.1 (6.4)	5.2 (1.8)	−19.4 (0.11)

**Table 7:** Results of compliance evaluations in terms of the *H*‐field RL (i.e., ) and BR (i.e., ) from IEEE Std. C95.1‐2019 according to different procedures for a user/bystander in zone 1, where is the incident *H*‐field measured. The Tier 3 assessment here is based on the *H*‐field gradient along the y axis (i.e., from the front to the back of the hob).

	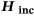 @30 cm/ 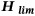	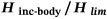	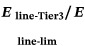	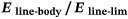
	(IEC 62233)	(IEC 63184 Tier 2)	(IEC 63184 Tier 3)	(IEC 63184 Tier 4)
Device	[dB] (unitless)	[dB] (unitless)	[dB] (unitless)	[dB] (unitless)
A	−40.7 (9.2 $\times \;10^{-3}$)	−7.0 (0.45)	−7.0 (0.45)	−12.9 (0.23)
B	−39.7 (1.0 $\times \;10^{-2}$)	−12.4 (0.24)	−12.4 (0.24)	−15.7 (0.16)
C	−42.1 (7.9 $\times \;10^{-3}$)	−12.0 (0.25)	−12.0 (0.25)	−17.7 (0.13)
D	−46.8 (4.6 $\times \;10^{-3}$)	−1.7 (0.82)	−1.9 (0.80)	−25.4 (5.4$\;\times \;10^{-2}$ )

**Table 8:** Results of compliance evaluations in terms of the *H*‐field RL (i.e., ) and BR (i.e., ) of ICNIRP 1998 according to different procedures for a user hand in zone 3, where ${\;\text{H}\;}_{\text{inc}}$ is the incident *H*‐field measured. The Tier 3 assessment here is based on the H‐field gradient along the z axis (i.e., from the bottom to the top of the hob).

	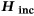 @30 cm/ 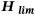	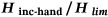	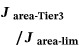	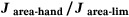
	(IEC 62233)	(IEC 63184 Tier 2)	(IEC 63184 Tier 3)	(IEC 63184 Tier 4)
Device	[dB] (unitless)	[dB] (unitless)	[dB] (unitless)	[dB] (unitless)
A	−10.4 (0.30)	59.6 (955)	47.2 (229)	18.2 (8.1)
B	−9.5 (0.33)	60.4 (1.0 $\times \;10^{3}$)	45.4 (186)	21.2 (11)
C	−11.9 (0.25)	63.8 (1.5 $\times \;10^{3}$)	50.1 (320)	23.0 (14)
D	−16.5 (0.15)	40.7 (108)	25.4 (19)	−2.5 (0.75)

**Table 9:** Results of compliance evaluations in terms of the *H*‐field RL (i.e., ) and ${\;\text{E}\;}_{\text{cube}}$ BR (i.e., ${\;\text{E}\;}_{\text{cube‐lim}}$) of ICNIRP 2010 according to different procedures for a user hand in zone 3, where is the incident *H*‐field measured. The Tier 3 assessment here is based on the *H*‐field gradient along the z axis (i.e., from the bottom to the top of the hob).

	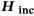 @30 cm/ 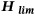	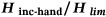	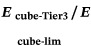	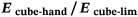
	(IEC 62233)	(IEC 63184 Tier 2)	(IEC 63184 Tier 3)	(IEC 63184 Tier 4)
Device	[dB] (unitless)	[dB] (unitless)	[dB] (unitless)	[dB] (unitless)
A	−22.9 (7.2 $\times \;10^{-2}$)	47.2 (229)	36.2 (65)	5.3 (1.8)
B	−21.9 (8.0 $\times \;10^{-2}$)	47.9 (248)	34.3 (52)	6.3 (2.1)
C	−24.3 (6.1 $\times \;10^{-2}$)	51.4 (372)	39.0 (89)	5.1 (1.8)
D	−29.0 (3.5 $\times \;10^{-2}$)	28.2 (26)	14.3 (5.2)	−20.5 (9.4 $\times \;10^{-2}$)

**Table 10:** Results of compliance evaluations in terms of the *H*‐field RL (i.e., ) and ${\;\text{E}\;}_{\text{line}}$ BR (i.e., ) of IEEE Std. C95.1‐2019 according to different procedures for a user hand in zone 3, where is the incident *H*‐field measured. The Tier 3 assessment here is based on the *H*‐field gradient along the z axis (i.e., from the bottom to the top of the hob).

	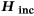 @30 cm/ 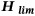	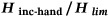	${\;\text{E}\;}_{\text{line‐Tier3}}∕{\;\text{E}\;}_{\text{line‐lim}}$	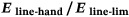
	(IEC 62233)	(IEC 63184 Tier 2)	(IEC 63184 Tier 3)	(IEC 63184 Tier 4)
Device	[dB] (unitless)	[dB] (unitless)	[dB] (unitless)	[dB] (unitless)
A	−40.7 (9.2$\;\times \;10^{-3}$)	14.5 (5.3)	14.3 (5.2)	−12.2 (0.25)
B	−39.7 (1.0$\;\times \;10^{-2}$)	15.3 (5.8)	14.4 (5.3)	−10.0 (0.32)
C	−42.1 (7.9$\;\times \;10^{-3}$)	18.7 (8.6)	18.3 (8.2)	−7.7 (0.41)
D	−46.8 (4.6$\;\times \;10^{-3}$)	−4.4 (0.60)	−5.5 (0.53)	−35.1 (1.8$\;\times \;10^{-2}$)

Although all appliances tested are considered to be of the same power class, the exposures of users/bystanders of the hobs differ largely by a factor of >20 (26 dB, see the fourth column of Tables 5, 6, 7, 8, 9, 10). One of the main findings is that the exposure is mainly a function of power, coil size, and proximity to the active coil (see Table 2).

With respect to demonstration of compliance, our study confirms and further strengthens the findings of (Christ et al. 2012) that the currently applied procedures for “Test conditions for induction hobs and hotplates” defined in the product standards IEC 62233 (IEC International Electrotechnical Commission 2005) and EN 62233 (CENELEC, European Committee for Electrotechnical Standardization 2008) are not suited to ensure safety and compliance with the BR for induction hobs. In IEC 62233 and EN 62233, the test requirement is simplified to *H*‐field measurements at a distance of 30 cm, which is only very poorly correlated with actual exposure (Gryz et al. 2020; Kitajima et al. 2022). The potential underestimation of the exposure is massive and may exceed a factor of 30 when testing against the exposure limits from (ICNIRP 1998; IEEE 2019). Although the current procedures greatly underestimate maximum exposure, no hazards have been reported to our knowledge. However, this may be because commercial hobs have not yet fully exploited the limits of the safety standard.

On the other hand, all four hobs tested fail in the compliance evaluation where the incident field at the location of users/bystanders or hands is compared with the RL limits (Tier 2 of IEC 63184 (IEC International Electrotechnical Commission 2021)) due to the very high local incident fields. As the incident fields decay steeply as a function of distance, the Tier 2 approach overestimates the induced fields for the investigated scenarios by factors of greater than 15 (24 dB) (ICNIRP 1998), 60 (36 dB) (ICNIRP 2010), and 15 (24 dB) (IEEE 2019) for users/bystanders, and by factors of greater than 140 (43 dB) (ICNIRP 1998), 270 (49 dB) (ICNIRP 2010), and 34 (31 dB) (IEEE 2019) for exposure of the hand. The extent of overestimation was quantified by comparing the results in the second and fourth columns of Tables 5, 6, 7, 8, 9, 10.

The most accurate assessment is the dosimetric evaluation (Tier 4 of IEC 63184 (IEC International Electrotechnical Commission 2021)), the feasibility of which has been demonstrated in this study. However, a Tier 4 assessment is very demanding and costly. Furthermore, when the limits of ICNIRP 1998 (ICNIRP 1998) are applied, a better definition of where to evaluate 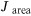
—that is, whether only in the central nervous system or in all tissues—needs to be specified by the regulators. If the limit is applied to all tissues, the potential nerve stimulation hazards will be overestimated. For example, the maximum 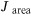 in 9‐month‐pregnant Ella would be up to 4 dB higher when the amniotic fluid is included.

The overestimation of exposure is lower when assessed according to Tier 3 of IEC 63184, but the reduction is not sufficient. However, refinement of Tier 3 for applicability to induction hobs should be the next step in standardization for conservative and resource‐ and time‐efficient compliance testing.

With respect to exposures in terms of contact current and incident *E*‐field, IEC 60990 (IEC International Electrotechnical Commission 2016) can be applied directly to evaluate compliance without the need for further refinement.

The important conclusions of this study are that the exposure of induction hobs can be significantly reduced by employing small heating coils and that the IEC 62233 standard (IEC International Electrotechnical Commission 2005) urgently requires revision to ensure the safety of induction hobs.

## Conflicts of Interest

Niels Kuster is the founder of Schmid & Partner Engineering AG (SPEAG) and ZMT Zurich MedTech AG (ZMT). He is also a minority shareholder of NFT Holding AG, which owns shares in SPEAG and ZMT. Sven Kühn serves on SPEAG's board of directors, while Jingtian Xi leads the team developing MAGPy. SPEAG specializes in measurement equipment for assessing exposure in the near field of electromagnetic (EM) sources, while both SPEAG and ZMT provide simulation tools for determining incident and induced fields.

## Data Availability

The data that support the findings of this study are available from the corresponding author upon reasonable request.
